# Application of a robust MALDI mass spectrometry approach for bee pollen investigation

**DOI:** 10.1007/s00216-024-05368-9

**Published:** 2024-06-15

**Authors:** Chiara Braglia, Daniele Alberoni, Diana Di Gioia, Alessandra Giacomelli, Michel Bocquet, Philippe Bulet

**Affiliations:** 1 Dipartimento di Scienze e Tecnologie Agro-Alimentari (DISTAL), Università di Bologna, Viale Fanin 42, 40127 Bologna, Italia; 2 Unione Nazionale Associazioni Apicoltori Italiani (UNA API), Via Pietro Boselli 2, Firenze, Italia; 3 Apimedia, 82 Route de Proméry, Pringy, 74370 Annecy, France; 4 CR, University Grenoble Alpes, IAB Inserm 1209, CNRS UMR5309, 38000 Grenoble, France; 5 Plateforme BioPark of Archamps, 74160 Archamps, France

**Keywords:** Mass spectrometry, Molecular mass fingerprint, Trifluoroacetic acid, Acetonitrile, Machine learning model, Plant biodiversity

## Abstract

Pollen collected by pollinators can be used as a marker of the foraging behavior as well as indicate the botanical species present in each environment. Pollen intake is essential for pollinators’ health and survival. During the foraging activity, some pollinators, such as honeybees, manipulate the collected pollen mixing it with salivary secretions and nectar (corbicular pollen) changing the pollen chemical profile. Different tools have been developed for the identification of the botanical origin of pollen, based on microscopy, spectrometry, or molecular markers. However, up to date, corbicular pollen has never been investigated. In our work, corbicular pollen from 5 regions with different climate conditions was collected during spring. Pollens were identified with microscopy-based techniques, and then analyzed in MALDI-MS. Four different chemical extraction solutions and two physical disruption methods were tested to achieve a MALDI-MS effective protocol. The best performance was obtained using a sonication disruption method after extraction with acetic acid or trifluoroacetic acid. Therefore, we propose a new rapid and reliable methodology for the identification of the botanical origin of the corbicular pollens using MALDI-MS. This new approach opens to a wide range of environmental studies spanning from plant biodiversity to ecosystem trophic interactions.

**Graphical Abstract:**

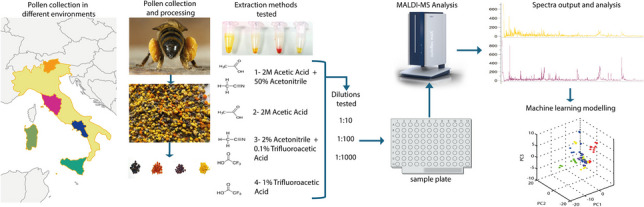

**Supplementary Information:**

The online version contains supplementary material available at 10.1007/s00216-024-05368-9.

## Introduction

Identification of pollen is crucial in different disciplines ranging from plant taxonomy and their evolutionary relationships with pollinators, to allergies and paleobotany studies [1]. In particular, pollinators are strongly dependent on pollen for their survival, and they can collect from a wide range of pollen sources, also covering considerable areas [2]. Therefore, pollen can be exploited by researchers for different purposes such as environmental pollution monitoring [3, 4], and also as a tool for vegetation surveys [5]. One of the major concerns in the research on plant-pollinator networks is pollen composition and nutritional properties, which can greatly impact the whole ecosystem, especially pollinators. The pollen’s nutritional potential is a key factor for pollinators’ survival and health [6], and is strictly related to each ecosystem flower species [7, 8] and to the space-temporal shifts during the season [9, 10]. Anthropogenic activities, climate changes, biodiversity decline, and the spread of invasive species and diseases have shown a dramatic impact on plant physiological state and, consequently, on pollen availability and nutritional profile [11–14].

An unbalanced pollen diet not providing the right amount and quality of proteins, as well as vitamins and lipids [15, 16], has a negative impact on the honeybee health and development [9, 17]. Indeed, the nutritional richness of pollen is crucial and has effects on (i) honeybee survival and metabolism [18–21], (ii) immunity and stress resistance [6, 22, 23], (iii) pathogen tolerance [24, 25], and (iv) sensitivity to agrochemicals [26–28]. Specifically, pollen consumption provides essential amino acids necessary for hypopharyngeal glands, ovaries, fat body development [29], and immune cell diversification [30, 31]. Considering that there is no plant species capable of providing all the amino acids [32], the importance of a varied and balanced diet cannot be underestimated. However, the impoverishment of resources worsened by the spread of monocultures in intensive agriculture, making a nutrient and complete diet difficult to obtain [33].

Matrix-assisted laser desorption ionization–mass spectrometry (MALDI-MS) is recognized as a robust technique in the identification of microorganisms in clinical diagnostics [34]. This technique is also known as MALDI Biotyping. In 2021, Houdelet and colleagues used MALDI Biotyping to identify the species and the geographical origin of *Nosema* spores [35]. To date, the use of MALDI-MS on pollen has been limited to the investigation of lipid and protein content involved in the development of allergies [36–38]. Despite this, in recent years, the efficiency of MALDI-MS in the identification of botanical species within collected environmental pollens has been demonstrated [13, 39–43]. MALDI-MS is a destructive technique that provides information on the molecular mass of each ionized molecule detected and gives access to a semi-quantitative analysis of samples, differently from IR and RAMAN spectroscopy that identify functional groups only. Moreover, MALDI-MS is not affected by colored matrices, such as pollens. Therefore, from these considerations, we propose a methodology based on molecular mass fingerprint profiles (MFPs) of pollen’s proteins, with MALDI-MS.

In this work, the pollen collected by foraging honeybees from different plant species and geographical locations was used to develop a new rapid and reliable methodology for the identification of the botanical origin of the foraged pollens using MALDI-MS. The identification of the botanical origin of corbicular pollens is a preliminary step for the evaluation of pollen trophic effect on pollinators and, consequently, allows understanding of possible alterations in the pollinator foraging behavior.

## Materials and methods

### Pollen collection and palynological analysis

Bee pollen balls were collected by five beekeeping farms located in five Italian regions characterized by different latitudes, climate conditions, and plant resources (Campania, Sardegna, Sicilia, Trentino-Alto Adige, and Toscana) using traditional pollen traps. The collected polyfloral pollen was divided into monofloral pollen samples through visual morphological analyses (e.g., color). Moreover, palynological analysis was carried out for each monofloral pollen (PianaRicerca Srl, Castel San Pietro Terme, Bologna, Italy) on five different balls belonging to the same subset, to confirm the botanical origin (Table S1). The obtained optical microscope images were classified according to the period of collection, the region, and the climate, and were compared to those present in the international database PalDat—Palynological Database [44].

### MALDI-MS methods for analysis on pollen

#### Pollen preparation

Monofloral pollen samples previously stored at − 20 °C were dried prior solvent extraction and analysis by MALDI-MS. A first set of one bee pollen ball (approx. 0.012 g) was directly extracted with 1 µL formic acid on MTP 384 steel plate and matrix solution (see below) directly added to the steel plate. Moreover, single-pollen balls were processed with four different extraction solutions using different solvents dissolved, when necessary, in ultrapure water (MilliQ water, Millipore, Billerica, USA). The four tested solvent solutions were as follows: (1) 2 M pure acetic acid (AA) added with 50% acetonitrile (ACN); (2) 2 M pure AA; (3) a mixture of 2% ACN and 0.1% trifluoroacetic acid (TFA); (4) 1% TFA. A total volume of 50 µL of each solvent solution was added to a single-pollen ball.

For each pollen extracted with solvents, two different disruption methods were tested: a sonication cycle of 15 min at 60 Hz and a shaken approach at 800 rpm for 1 h at 4 °C. The obtained extracts were diluted 10, 100, and 1000 times in 2% ACN in 0.1% TFA. Dilutions were spotted on a MTP 384 MALDI polished target plate (Bruker Daltonics, Germany) following a dry droplet sample preparation. Briefly, 1 µL of each dilution and 1 µL of alpha-cyano-4-hydroxycinnamic acid matrix (4-CHCA) solution (15 mg/mL) prepared in 70% ACN with 2.5% TFA in ultrapure water (v:v) were spotted and dried at room temperature under a soft vacuum. The molecules extracted from pollen for each condition were analyzed in MALDI-MS in triplicate.

#### Data acquisition

MALDI molecular mass fingerprints (MFPs) were acquired in a positive ion linear mode using an AutoFlex III—Smartbeam® mass spectrometer (Bruker Daltonics, Germany) [45, 46]. An external mass spectrometer calibration was achieved using the PepMix and ProtMix (Bruker Daltonics) standard calibration kits covering the dynamic range of 700 and 8560 Da. Spectra were acquired between the dynamic range of 600–18,000 Da using FlexControl v4.0 Software (Bruker Daltonics, Germany). A global attenuator offset of 60% and attenuator range of 5% with 200 Hz laser frequency, 70% of laser power, and 1000 accumulated laser shots/pollen spectrum were set. A potential difference of 1.5 kV and linear analog offset of 50.0 mV were set up and a suppression gate up to *m/z* 600 to prevent detector saturation. Sensitivity was set up at 100 mV.

#### Data post-processing, machine learning model, and statistical analysis

MALDI-MS data were visualized with FlexAnalysis v3.4 Software (Bruker Daltonics, Germany) and post-processed and analyzed (PCA and modeling) using ClinProTools™v2.2 Software (Bruker Daltonics, Germany) [47]. Spectral smoothing and baseline subtraction were performed, followed by the calculation of the total averaged spectra based on area calculations and a signal-to-noise ratio (S/N) of 5 for peak picking. Principal component analysis and machine learning mode were performed on the five major representative botanical families (*Asteraceae*, *Boraginaceae*, *Fagaceae*, *Leguminosae*, and *Rosaceae*). Specifically, a supervised neural network (SNN) algorithm was used for model development, with the following parameters: resolution 800, minimal baseline width 10%, and number of prototypes 5. For more information, refer to the specific section in supplementary materials “Appendix 1.”

## Results

The solvent-extracted pollen molecules used for the calibration test of the presented methodology derive from *Quercus* spp., since this pollen is widely distributed in all the regions considered in our study.

### A rapid, reliable, and economic methodology for MALDI-MS analysis on bee pollen

**Fig. 1:**
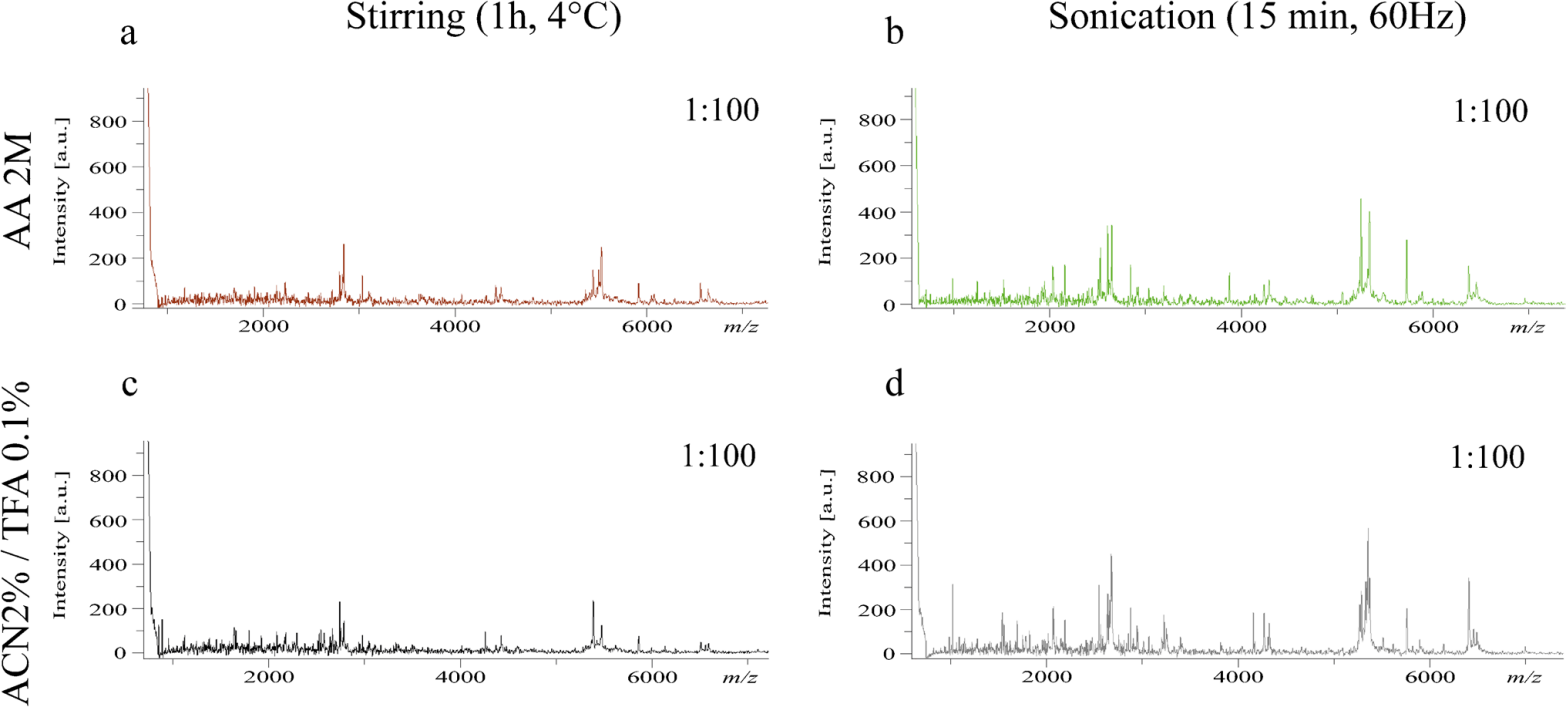
Mass spectra obtained from the best extraction conditions and according to the dilution factor of the crude extracts 1:100. Spectra were cut between range *m/z* 600–7000 to highlight peaks of interest. **a** Spectra of pollen extracted in AA 2 M and stirred (1 h, 4 °C). **b** Spectra of pollen extracted in AA 2 M and sonicated; **c** spectra of pollen extracted in ACN 2%/TFA 0.1% and stirred (1 h, 4 °C). **d** Spectra of pollen extracted in ACN 2%/TFA 0.1% and sonicated. [a.u.] stands for arbitrary unit

The most reliable results in terms of reproducibility, spectral intensity, and complexity were obtained at the 100-fold dilution for each tested method (tested from 10 to 1000-fold dilution factors). At first glance, the different extracting conditions tested (four solutions, two mechanical methods “paragraph 2.1”), have revealed interesting outputs (Fig. 1a–d and Fig. S1a–S1h). However, it was possible to identify two preferable protocols in terms of peak abundance and intensity. Specifically, the 100-fold dilutions after 15 min of sonication in 2 M AA and 2% ACN and 0.1% trifluoroacetic acid (0.1% TFA) (Fig. 1b and d) result in the highest level of output. A detection of 185 and 205 ions at *m/z* was recorded using 2 M AA and 2% ACN and 0.1% TFA, respectively.

### Palynological analysis and pollen classification

A total of 23 plant genera were identified, belonging to 16 botanical family: *Cistaceae* (*Cistus* sp., *Helianthemum* sp.), *Boraginaceae* (*Echium* sp., *Borago* sp.), *Leguminosae* (*Hedysarum* sp., *Trifolium* sp.), *Asteraceae* (*Compositae* group), *Fagaceae* (*Quercus* sp., *Castanea* sp.), *Rosaceae* (*Rubus* sp., *Crataegus* sp., *Amygdaloideae* group), *Cornaceae* (*Cornus* sp.), *Salicaceae* (*Salix* sp.), *Ericaceae* (*Erica* sp.), *Anacardiaceae* (*Pistacia* sp.), *Brassicaceae* (*Sinapis* sp.), *Dipsacaceae* (*Knautia* sp.), *Sapindaceae* (*Aesculus* sp.), *Papaveraceae*, (*Papaver* sp.), *Arecaceae* (*Trachycarpus* sp.), and *Asparagaceae* (*Asparagus* sp.). Moreover, eight pollen were discriminated at the species level, in particular *Cistus incanus*, *C. salvifolius*, *Hedysarum coronarium*, *Trifolium repens*, T*. hibridum*, *T. incarnatum*, *Cornus sanguinea*, and *Asparagus officinalis*. The pollen ball classification and morphological characteristics from palynological analysis are reported in Table 1 and Table S2, respectively.

**Table 1:** Report on the pollens used and their relative botanical classification by family and genus (species where provided) derived from palynological analysis and the relative quantity and origin

Family	Botanic group/genus/specie	No. of pollen samples	Campania	Sardinia	Sicily	Tuscany	Trentino
*Cistaceaae*	*Cistus incanus*	2		**X**			
	*Cistus salvifolius*	1		**X**			
	*Helianthemum sp.*	1					**X**
*Boraginaceae*	*Echium*	3		**X**			
	*Borago*	1	**X**				
*Leguminosae*	*Hedysarum coronarium*	1	**X**				
	*Trifolium repens*	1					**X**
	*Trifolium hibridum*	1		**X**			
	*Trifolium incarnatum*	1		**X**			
*Asteraceae*	Compositae T (liguliflore)	2				**X**	**X**
	Compositae S (thistle)	2		**X**	**X**		
*Fagaceae*	*Quercus*	5	**X**	**X**	**X**	**X**	
	*Castanea*	1					**X**
*Rosaceae*	*Rubus*	4		**X**	**X**		**X**
	*Crataegus*	1	**X**				
	*Amygdaloideae*	2		**X**			**X**
*Cornaceae*	*Cornus sanguinea*	2	**X**				
*Salicaceae*	*Salix*	1					**X**
*Ericaceae*	*Erica*	1				**X**	
*Anacardiaceae*	*Pistacia*	1		**X**			
*Brassicaceae*	*Sinapis*	1					**X**
*Dipsacaceae*	*Knautia*	1	**X**				
*Sapindaceae*	*Aesculus*	1	**X**				
*Papaceraceae*	*Papaver*	1	**X**				
*Arecaceae*	*Trachycarpus*	1	**X**				
*Asparagaceae*	*Asparagus officinalis*	1	**X**				

### MFPs recorded by MALDI-MS well discriminate bee pollen from different botanical families

A total of 40 different bee-collected pollens were extracted using an ultrasound system and AA 2 M as solvent (previously described), in order to validate a fast and accessible methodology. A total of 132 spectra were recorded and manually sorted at the family level to allow a first robust comparison using PCA. *Asteraceae* (*n* = 4), *Boraginaceae* (*n* = 4), *Fagaceae* (*n* = 6), *Leguminosae* (*n* =4), and *Rosaceae* (*n* = 7) were the most recurrent families with the highest number of pollen samples and then used for the analysis. *Cistaceae* (*n* = 3), *Cornaceae* (*n* = 2), *Ericaceae* (*n* = 1), *Salicaceae* (*n* = 1), *Anacardiaceae* (*n* = 1), *Brassicaceae* (*n* = 1), *Arecaceae* (*n* = 1), *Dipsacaceae* (*n* = 1), *Papaveraceae* (*n* = 1), and *Sapindaceae* (*n* = 1) were in an inconsistent number to allow robust analysis and were excluded from the PCA. The total number of ions was between 97 and 204 with an average intensity of 93.01. The highest peak intensity was recorded between molecular ions at *m/z* between 700 and 2000 and *m/z* 3000 and 5000. The first visual analysis using flexAnalysis tools showed typical and homogeneous spectra for each family considered. Variations among pollen from the same family examples are reported in Fig. S2a and S2b.

Principal component analysis (PCA) was applied to compare the classification of pollen species and confirmed an evident family clustering, especially for *Asteraceae* and *Boraginaceae* for PC1 *vs* PC2 and PC1 *vs* PC3, but also for *Rosaceae* and *Fagaceae*. Leguminosae did not show any family clustering, but they spread along the y axis in particular in the output PC1 *vs* PC2 and PC1 *vs* PC3. Among 100 peaks manually selected, Kruskal–Wallis test was considered for 91 non-normal distributed peaks individuated by PCA analysis in *m/z* range of 757 and 6925. Component comparison resulted highly significative for 56 peaks (*p* < 0.001) and widely significative for 24 peaks (*p* < 0.05).

**Fig. 2:**
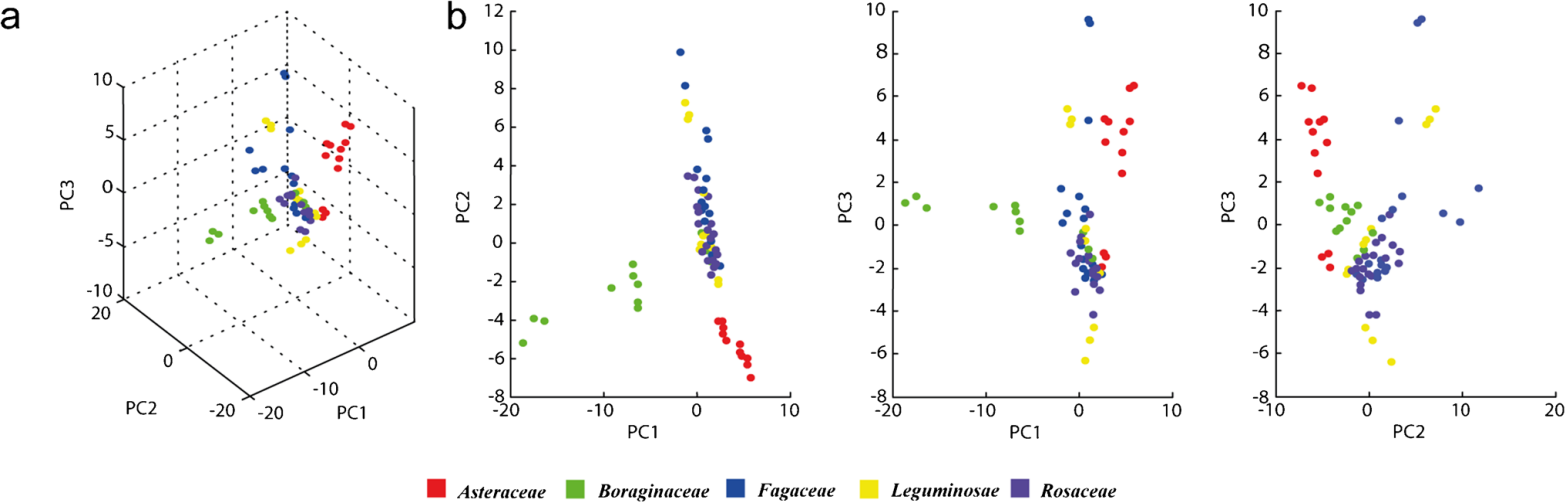
Principal component analysis (PCA) output: three-dimensional plot considering principal components 1, 2, 3 (**a**) and bidimensional plot considering each principal component comparisons (**b**). Dots represent different families as follows: (red) *Asteraceae*; (green) *Boraginaceae*; (blue) *Fagaceae*; (yellow) *Leguminosae*; (purple) *Rosaceae.* The overall variation is 39% for the 3 axes (18% for axe 1 (PC1), 11% for axe 2 (PC2), 10% for axe 3 (PC3))

Classes were discriminated by a minimum of 11 peaks of each extracted pollen molecule. Mass average, standard deviation, and *p*-value for each considered peak are reported in Table S3. Deviations between the same groups are visualized in the generated graph (Fig. 2a–b) and might indicate different origins, genuses, or species, as well as occurred for spectra. Moreover, to support the botanical family discrimination, we have detected recurrent peaks among those considered in PCA analysis that are significantly related to each of the botanical family analyzed. Specifically, 24 peaks are unique for the botanical family analyzed: 5 for *Asteraceae*, 6 for *Boraginaceae*, 7 for *Fagaceae*, 3 for *Leguminosae*, and 3 for *Rosaceae* (Table 2).

**Table 2:**
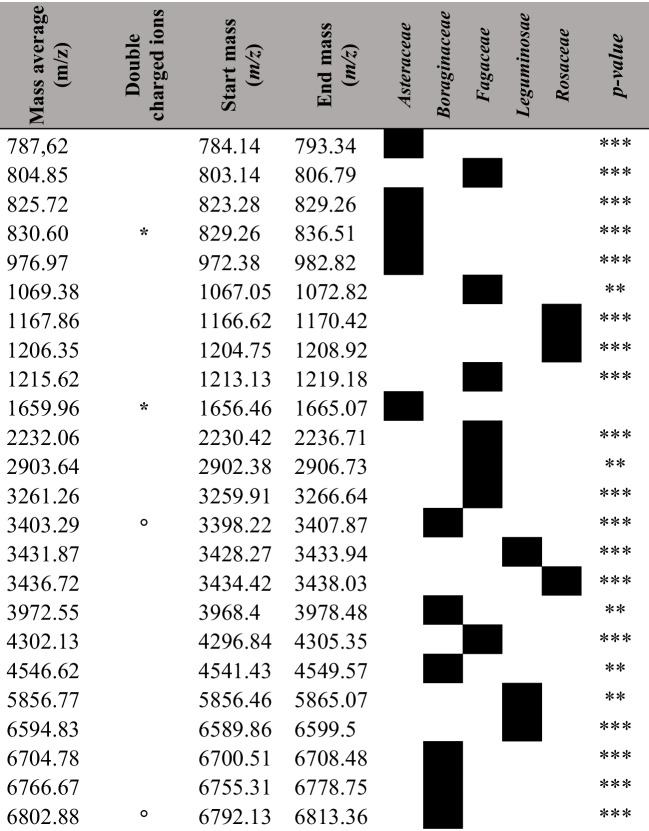
Table shows SNN algorithm classified peaks’ average, start, and end mass. Class discriminations and the relative PCA *p*-value are reported. Double-charged ions are indicated with * and °. *p*-value; ** *p* > 0.05; *** *p* > 0.001

### A machine learning model for easy bee pollen classification

A novel SNN algorithm model was generated considering all the single spectra intensity from the same five family classes (class 1, *Asteraceae*; class 2, *Boraginaceae*; class 3, *Fagaceae*; class 4, *Leguminosae*; and class 5, *Rosaceae*). The irrelevant spectra that did not pass the required intensity and signal resolution were excluded, and then a manual peak curation was performed on reliable peaks. A cluster of 25 automatically selected peaks by machine learning was used, after manual check, with a cycle upper limit of 2000 and five prototypes (Fig. S1). A total of 22 peaks were significatively discriminated as possible indicators of pollen origin with an overall cross-validation of 80.81% and cross-capability of 90.97%. Detected discriminant molecular double-charged ions were considered (*m/z* 830.4 *vs* 1659.96 and 3403.29 *vs* 6802.88, respectively) and no sodium or potassium adducts were individuated in highlighted peaks. Cross-validation and recognition capability values for each class and classified peaks’ mass average, start, and end mass are reported in Tables 2 and 3.

**Table 3:** The table shows cross-validation and recognition capability percentage values for each class analyzed and overall

Class	Family	Cross-validation (%)	Recognition capability (%)
Class 1	*Asteraceae*	92.00	100.00
Class 2	*Boraginaceae*	89.47	84.62
Class 3	*Fagaceae*	79.41	83.33
Class 4	*Leguminosae*	50.00	91.67
Class 5	*Rosaceae*	93.18	95.24
Overall		**80.81**	**90.97**

## Discussion

Considering the nutritional problems that pollinators are facing, triggered by climate change and anthropic activities, the development of a method that allows rapid identification of pollens harvested by bees is pioneering for environmental-based studies. This work aims at developing a safe, reproducible, sensitive, and fast protocol to analyze and identify the botanical origin of pollen balls collected by honeybees during their foraging activities based on the analyses of pollen-extracted molecules, using MALDI-MS.

We firstly tested pollen balls extracted with pure formic acid, as a direct solution; however, this method did not allow the collection of peaks. This is reminiscent of the recent research works [42], Lauer et al. [40], Lauer et al. [41] that demonstrated how a direct spotting of a single or few pollen grains (pollen dust) on a MALDI plate can result in the taxonomy differentiation and identification of pollens [40] with or without any chemical extraction such as the use of formic acid [42]. It is worth noting that in our experimental conditions, this methodology did not provide robust signal outputs for the different samples analyzed (data not reported). The negative results obtained when applying the methodologies described by Seifert et al. [42] and Laurel et al. [40] may be ascribed to the use of samples of pollen dust collected directly from plants, whereas we used pollen balls produced by pollinators. Some pollinators, especially honeybees, when collecting pollen, mix it with nectar and salivary secretions, to easily manipulate and shape it in balls, easily transportable in their corbiculae (pollen baskets) to the nesting site (for review, see [48]. These modifications induce molecular and structural changes in the pollen grains (high compactness) that make them improper for direct MALDI-MS analysis. Moreover, layer thickness and inhomogeneity of the MALDI sample preparation might negatively impact laser ionization efficiency [49–51]. Interestingly, Krause et al. [39] demonstrated that on *Ambrosia trifiga* pollen, MALDI-MS is applicable on a few pollen grains (~ 20–30, estimated to correspond to 10 µg of commercially available lyophilized pollen). However, this parameter might not be controlled easily, especially on pollen ball samples because of their sticky structure. This evidence suggests that to process bee pollen balls, a solvent extraction may improve the availability of molecular ions detectable by MALDI-MS. Therefore, for the first time, we propose a protocol to analyze by MALDI-MS the extracted pollen balls.

The different extraction procedures we tested were demonstrated effective in giving readable outputs with a high reproducibility between the different extraction methods. The recorded spectra were homogeneous and similar, confirming the validity of any proposed extraction methods. The best performances were obtained using a sonication disruption method (one cycle of 15 min at 60 Hz). Ultrasound-assisted solvent extraction is an already-known useful method to break the pollen wall in different kinds of analyses, e.g., in flame atomic absorption spectroscopy [52–54]. When applied to the different pollen ball samples investigated, MFPs obtained by MALDI-MS support the hypothesis that MALDI-MS represents a robust approach for bee pollen ball classification. In addition, extraction conditions using acetic acid at the concentration of 2 M proved to be as efficient as conventional solution of trifluoroacetic acids (TFA) without the disadvantages of TFA, a highly volatile and toxic acid.

The protocols proposed in this work showed robust results in terms of specificity and pollen botanical source clustering, confirming that MALDI-MS technology is applicable also to bee pollen balls. Our work showed typical spectra for each botanical essence (*Asteraceae*, *Boraginaceae*, *Fagaceae*, *Leguminosae*, and *Rosaceae*) collected from different geographical regions, whose clusters were deeply confirmed by PCA analysis. Taxonomic relationship reconstruction, classification, and identification in archaeobotanical applications [13, 39, 42, 43], as well as in human allergenic compound isolation and analysis in palynology [38, 55–57], have already been performed by MALDI-MS technology. Despite that, comparisons with previous works were not easily conducted due to unavailability of the databases, and/or lack of pollens of relevance for pollinators. The only exception was represented by the comparison between *Quercus* sp. pollen directly collected from plants [42] and *Quercus* sp. spectra obtained in our research, which showed a reliable spectrum overlapping that confirms the validity and reproducibility of our method.

It is worth highlighting that also differences in the MFPs acquired among pollens of the same botanical families were detected. Our hypothesis would be that such differences may be attributed to (i) genus and species variations within each family, (ii) different geographical origins, and (iii) different nutritional parameters (especially in lipid and protein content). The high intensity detected especially for low molecular ions between 700 and 900 *m/z* might highlight variations that can be attributed to non-peptidic components of the pollen such as lipids and/or sugars (reviewed by Zemski Berry et al. [58] and Leopold et al. [59]. This work did not focus on the specific type of molecules extracted, but it is a molecular mass fingerprinting strategy that targets the molecular ion fingerprint maps of pollen extracts. However, we hypothetically expected to detect lipids and phospholipids from 600 to 930 m*/z* according to Liang et al. [60] and Schiller et al. [61] phenolic extracts from 600 to 930 m*/z* according to Khadhri et al. [62], and small and medium-size proteins all over the range (600 to 18,000 m*/z*). Vitamins that range from 100 to 500 m*/z* [63], terpenes from 400 to 600 m*/z* [64], and alkaloids ranging from 100 to 500 m*/z* [65, 66] were out of range of detection. Similarly, it cannot be excluded that specific and sporadic ions might be linked to pollutants, such as agrochemicals or fertilizers. It is known that foraged contaminated pollen is one of the pesticide exposure routes affecting pollinators [67–71]. However, pesticide and agrochemical residues possess very low molecular masses (~ *m/z* 80–300) out of range in our tests [72–74]; therefore, this hypothesis cannot be confirmed.

## Conclusions

In this work, an efficient and reliable methodology for the identification of the botanical origin of bee pollen balls with MALDI-MS was described. Concerns about the safety of the solvent employed and time efficiency were positively addressed. We also demonstrated that the generation of specific models based on pollen diversity is possible avoiding time-consuming chromatographic techniques. Further investigations are required to expand the analysis on a wider set of pollen balls deriving from different botanical families, geographical origins, and exposed to different environmental factors, and use all these information to create a robust database for pollen analysis. Finally, this research wants to lay the basis for an innovative and holistic methodology able to determine, in addition to the botanical origin, the bee pollen balls’ nutritional value (e.g., sugar, lipids, and protein content) with MALDI-MS.

### Supplementary Information

Below is the link to the electronic supplementary material.

Supplementary file1 (PDF 1.57 MB)

## Data Availability

Data on MALDI mass spectrometry is available on AMS-Acta repository (repository of the University of Bologna) at the following DOI: 10.6092/unibo%2Famsacta%2F7717.

## Declarations

### Source of biological material

Honeybees’ corbiculae [Environmental].

### Statement on animal welfare

Not applicable. No honeybees were sacrificed for this research.
