# Supplementary material for: Application of a robust MALDI mass spectrometry approach for bee pollen investigation
Source: Anal Bioanal Chem. 2024 Jun 15;416(19):4315–24. doi: 10.1007/s00216-024-05368-9 (PMC11271380; doi:10.1007/s00216-024-05368-9)
Supplement: Supplementary file 1 — Supplementary file1 (PDF 1.57 MB) [file 216_2024_5368_MOESM1_ESM.pdf]

Supplementary materials for:

Application of a robust MALDI mass spectrometry approach for bee pollen investigation

Chiara Braglia[a], Daniele Alberoni[a*], Diana Di Gioia[a], Alessandra Giacomelli[b], Michel Bocquet[c], Philippe Bulet[d,e]

Affiliations:
a Dipartimento di Scienze e Tecnologie Agro-Alimentari (DISTAL), Università di Bologna, Viale Fanin 42, 40127, Bologna, Italia;

[b] Unione Nazionale Associazioni Apicoltori Italiani (UNA API), Via Pietro Boselli 2, Firenze, Italia

[c] Apimedia, 82 Route de Proméry, Pringy, 74370 Annecy, France

[d] CR, University Grenoble Alpes, IAB Inserm 1209, CNRS UMR5309, 38000 Grenoble, France
[e] Plateforme BioPark of Archamps, Archamps, France

***Corresponding Author:**
Daniele Alberoni (daniele.alberoni@unibo.it)

Supplementary results on the pollen extraction methods

Our experiment included bee pollen grains from five different Italian regions. In order to define the best experimental conditions to record robust and the most representative and distinguishable spectra for a pollen species, different conditions of extraction were tested, four different solutions: (1) 2M acetic acid 2M (2M AA) and 50% acetonitrile (50% ACN); (2) AA 2M; (3) 2% ACN and 0.1% trifluoroacetic acid (0.1% TFA); and (4) a solution of 1% TFA) and two mechanical extraction methods (stirring and ultrasonication). Moreover, 10-fold serial dilutions of the crude extracted material from the different bee pollen balls were evaluated (10, 100 and 1,000 times) to respect the most appropriate sample to matrix ratio.

The 10-fold dilution was only efficient after sonication despite low peaks intensity, while the 1000-fold dilution did not provide readable spectra, as well as direct positioned grains on steel plate and extracted with formic acid. Excluding the two best extraction methods results, the only other method that produced satisfying results was the 100-fold dilution after mechanical agitation (1h, 4°C) in 1% TFA, with 195 ions at m/z recorded at a significant relative intensity (Fig. S1g).

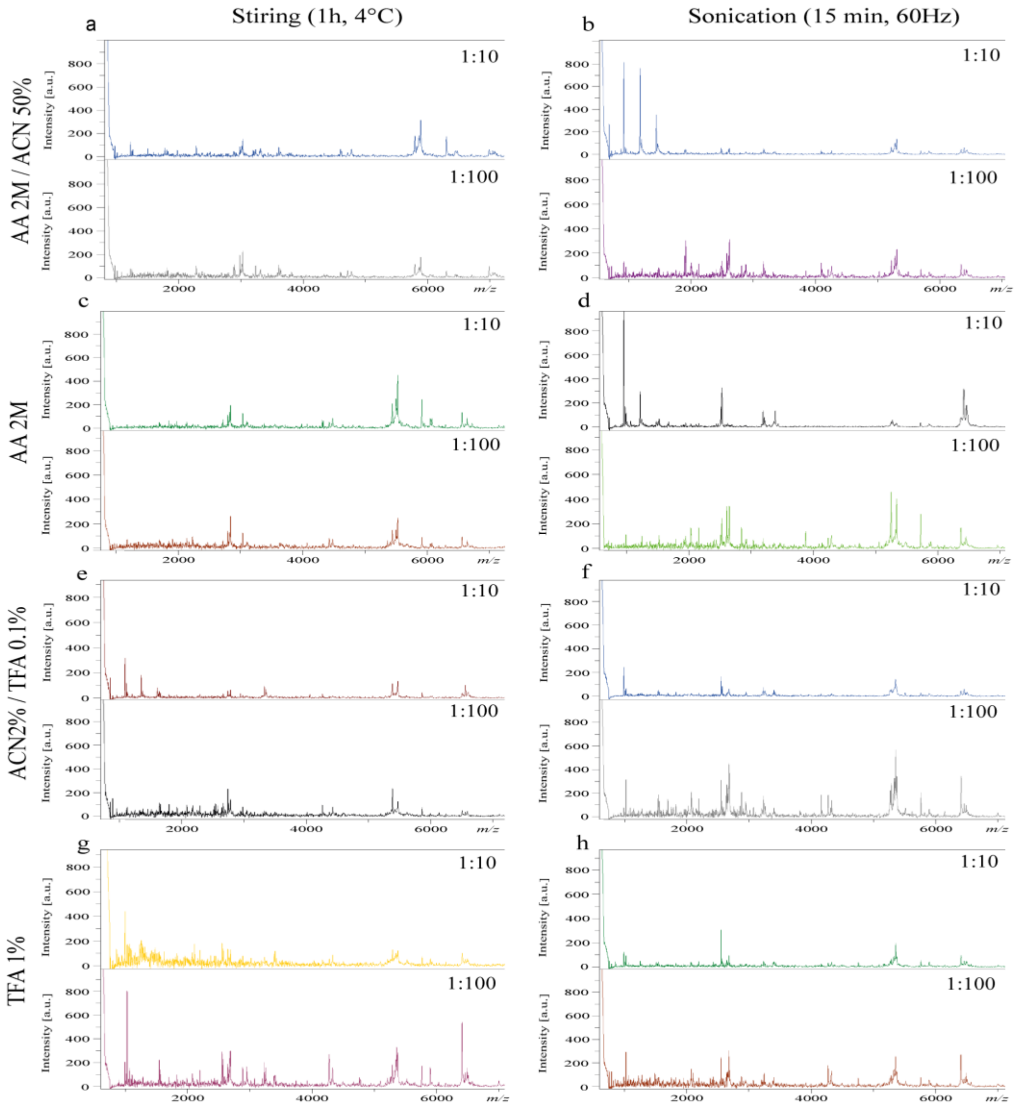

Fig. S1 Mass spectra obtained from the different extraction conditions and according to the dilution factor of the crude extracts. Spectra were cut between range *m/z* 600-7000 to highlight peaks of interest. Dilution 1:1000 did not provide useful spectra and were not reported in the figure. a) spectra of pollen extracted in AA 2M/ ACN 50% and stirred (1h, 4°C). b) spectra of pollen extracted in AA 2M/ ACN 50% and sonicated. c) spectra of pollen extracted in AA 2M and stirred (1h, 4°C). d) spectra of pollen extracted in AA 2M and sonicated; e) spectra of pollen extracted in ACN 2%/ TFA 0.1% and stirred (1h, 4°C). f) spectra of pollen extracted in ACN 2%/ TFA 0.1% and sonicated. g) spectra of pollen extracted in TFA 1% and stirred (1h, 4°C). h) spectra of pollen extracted in TFA 1% and sonicated. [a.u.] stand for arbitrary unit

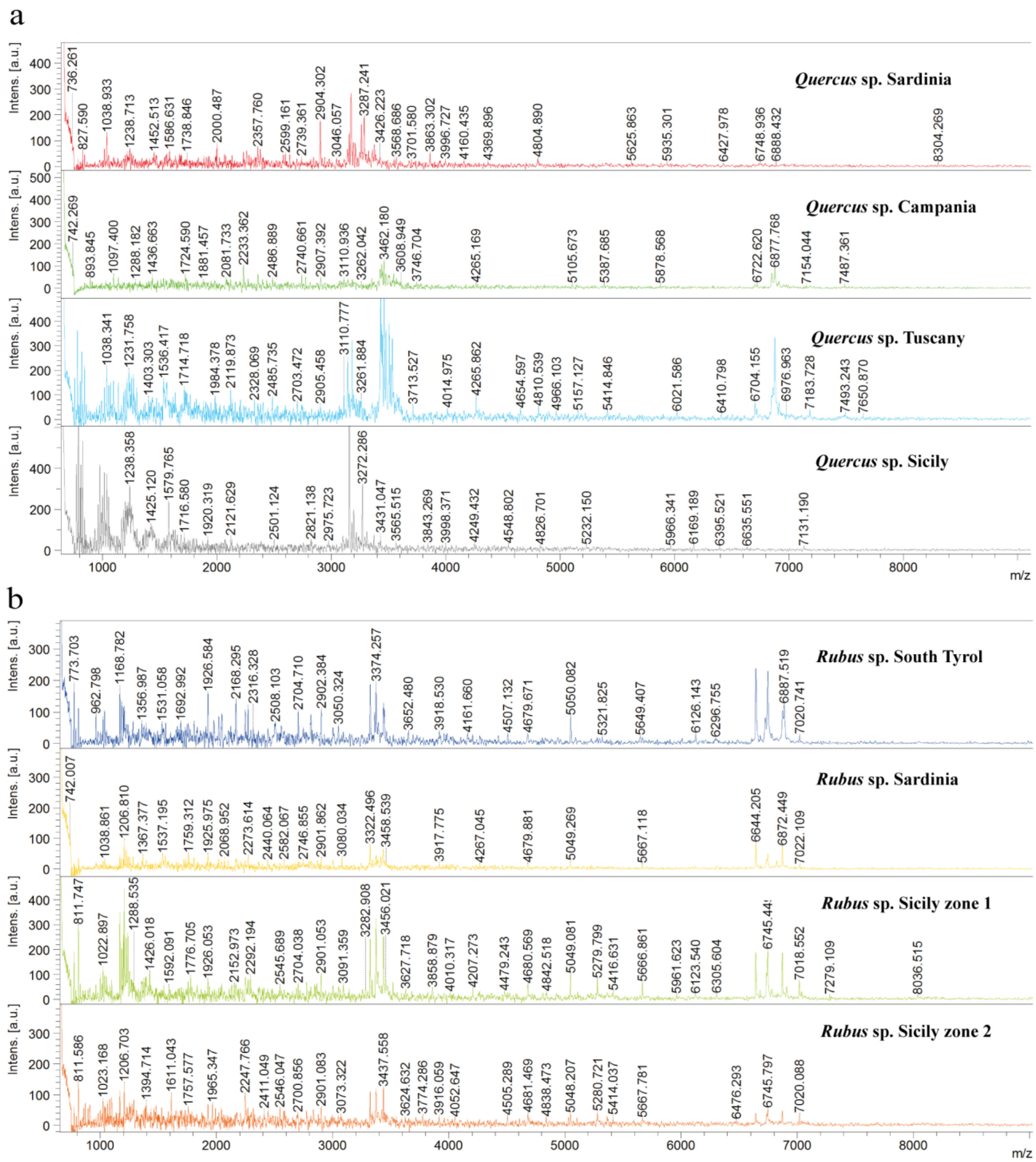

Fig. S2 (a) Comparison between *Quercus* sp. (*Fagaceae*) and (b) between *Rubus* sp. bee pollen balls from different Italian region origin (flexAnalysis tool visualization). Visualization of the comparisons were done using the ClinProTools was used. [arb.u.] and [a.u.] stand for arbitrary unit

Table S1. Pollen collection areas.

Code	Pollen origin (Region)	Pollen collection period	Geographic location (GPS coordinates)
**P11**	Sardinia	15/04/2023	40°15'11.7"N 9°09'51.0"E
**P12**	Sardinia	15/04/2023	
**P13**	Sardinia	15/04/2023	
**P14**	Campania	15/04/2023	41°01'36.1"N 14°54'28.7"E
**P15**	Campania	15/04/2023	
**P16**	Tuscany	15/04/2023	42°54'26.1"N 11°18'42.3"E
**P18**	Tuscany	15/04/2023	
**P21**	Tuscany	15/04/2023	
**P22**	Sicily	01/04/2023	38°09'15.9"N 13°17'35.5"E
**P23**	Sicily	01/04/2023	
**P25**	Sicily	01/04/2023	
**P26**	Sicily	01/04/2023	
**P27**	Sicily	01/04/2023	
**P28**	Sicily	01/04/2023	
**P31**	Sardinia	01/06/2023	40°15'11.7"N 9°09'51.0"E
**P32**	Sardinia	01/06/2023	
**P33**	Sardinia	01/06/2023	
**P34**	Campania	01/05/2023	41°01'36.1"N 14°54'28.7"E
**P35**	Campania	01/05/2023	
**P36**	Trentino	01/05/2023	45°53'28.1"N 10°44'09.5"E
**P37**	Trentino	01/05/2023	
**P50**	Sardegna	15/04/2023	40°15'11.7"N 9°09'51.0"E
**P51**	Sardegna	15/04/2023	
**P52**	Trentino	01/07/2023	45°52'18.1"N 10°39'01.2"E
**P53**	Trentino	01/07/2023	
**P54**	Trentino	01/07/2023	
**P56**	Trentino	01/07/2023	
**P57**	Trentino	01/07/2023	
**P58**	Trentino	01/07/2023	
**P59_1**	Sardinia	01/06/2023	40°15'11.7"N 9°09'51.0"E
**P61**	Sardinia	15/04/2023	
**P62**	Sardinia	15/04/2023	
**P63**	Sardinia	15/04/2023	
**P64**	Campania	15/05/2023	41°01'36.1"N 14°54'28.7"E
**P65**	Campania	15/05/2023	
**P69_1**	Sardinia	01/06/2023	40°15'11.7"N 9°09'51.0"E
**P69_2**	Sardinia	01/06/2023	

Table S2. Botanical classification for each pollen tested in each different experiment.

Code	Grains morphology	Botanical classification	Code	Grains morphology	Botanical classification
**P11**	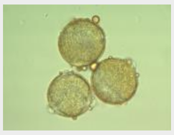	*Cistus incanus*	**P22**	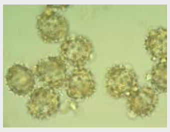	*Compositae T (liguliflore)*
**P12**	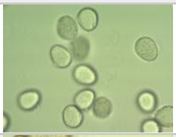	*Echium*	**P23**	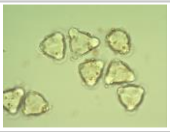	*Genista*
**P13**	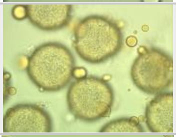	*Cistus monspeliensis*	**P24**	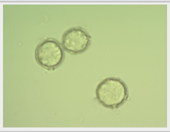	*Pistacia*
**P14**	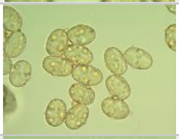	*Hedysarum coronarium*	**P25**	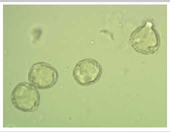	*Quercus*
**P15**	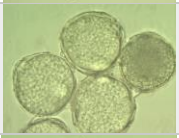	*Cornus sanguinea*	**P26**	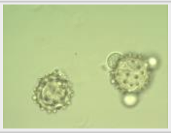	*Compositae S (cardi)*
**P16**	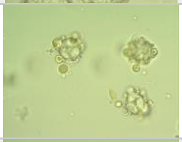	*Compositae T (liguliflore)*	**P27**	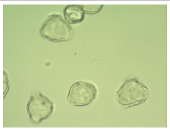	*Rubus*
**P17**	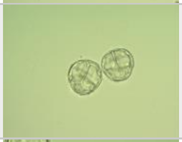	*Erica*	**P28**	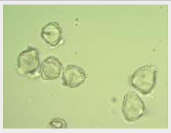	*Rubus*
**P18**	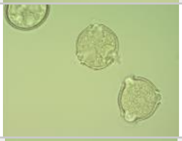	*Quercus*	**P31**	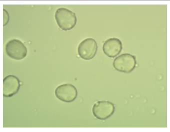	*Echium*
**P21**	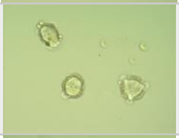	*Salix*	**P32**	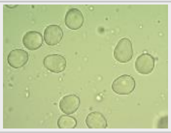	*Echium*
**P34**	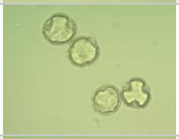	*Quercus*	**P56**	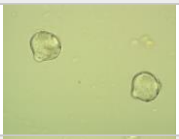	*Trifolium repens*
**P35**	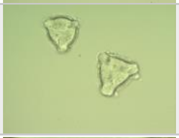	*Crataegus*	**P57**	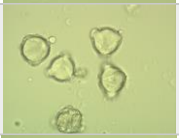	*Rubus*
**P36**	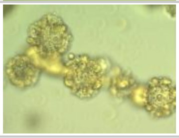	*Compositae T (liguliflore)*	**P58**	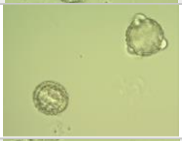	*Centaurea gruppo jacea*
**P37**	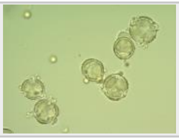	*Salix*	**P59_1**	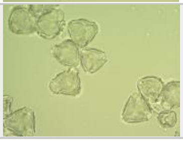	*Rubus*

**P50**	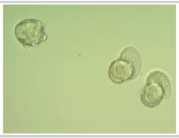	*Cruciferae*	**P61**	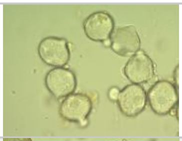	*Trifolium hibridum*
**P51**	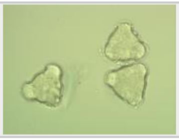	*Malus/Pyrus/Sorbus (Amygdaloideae)*	**P62**	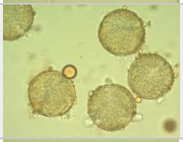	*Cistus incanus*
**P52**	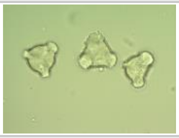	*Malus/Pyrus/Sorbus (Amygdaloideae)*	**P63**	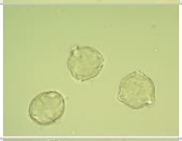	*Quercus*
**P53**	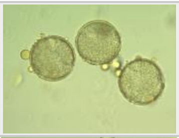	*Helianthemum*	**P64**	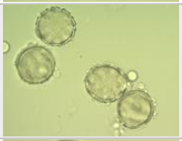	*Borago*
**P54**	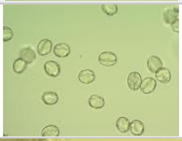	*Castanea*	**P65**	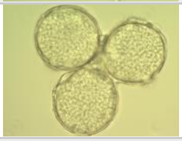	*Cornus sanguinea*
**P55**	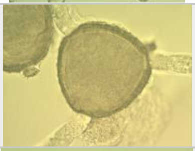	*Knautia*	**P66**	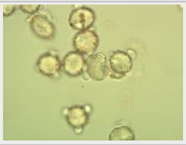	*Aesculus*
**P67**	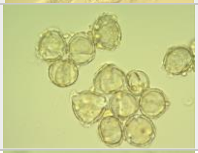	*Papaver*			
**P68**	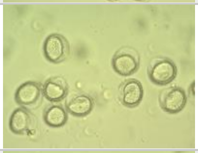	*Trachycarpus*			
**P69**	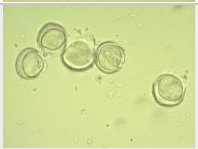	*Asparagus officinalis*			
**P69_1**	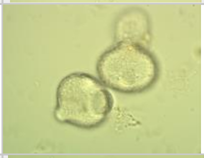	*Trifolium incarnatum*			
**P69_2**	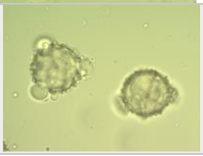	*Compositae S (cardi)*			

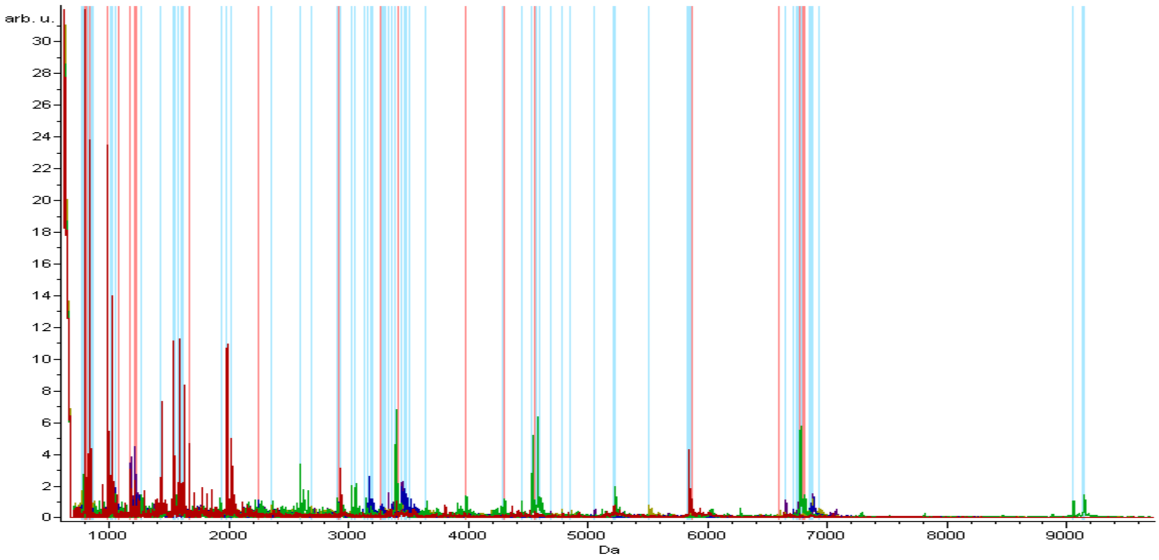

Figure S1. In blue, peaks considered for the PCA analysis and SNN Algorithm. In red, automatically assigned peaks from SNN Algorithm used for model generation.

Table S3. Table shows mass average intensity, standard deviation ad p-value for each considered peak. Averages in bold indicate the Class where the intensity is higher.

Mass (*m/z*)	Class1 (average)	Class2 (average)	Class3 (average)	Class4 (average)	Class5 (average)	*p-value*		StdDev Class1	StdDev Class2	StdDev Class3	StdDev Class4	StdDev Class5
787.62	**45.88**	0.38	3.04	0.39	0.52	0.00000252	***	25.91	0.4	4.87	0.37	0.48
804.85	1.04	0.58	**1.84**	0.68	0.28	0.0000378	***	0.78	0.25	1.74	0.54	0.19
810.85	**4.37**	0.41	1.79	0.91	2.82	0.00000399	***	3.42	0.22	2.76	0.92	1.61
825.72	**24.33**	0.57	2.34	0.83	0.69	<0.000001	***	13.6	0.4	2.94	0.56	0.43
830.6	**8.72**	0.64	0.98	0.56	0.56	0.0000203	***	4.77	0.16	1.04	0.29	0.31
842.98	0.89	0.98	**1.77**	1.12	0.69	0.000376	***	0.31	0.27	1.21	0.65	0.1
860.04	0.68	0.79	1.02	**1.54**	0.58	0.0000448	***	0.16	0.15	0.38	0.91	0.13
9048.6	0.16	**1.4**	0.2	0.19	0.18	0.000983	***	0.04	1.5	0.06	0.07	0.06
9138.34	0.14	**1.71**	0.2	0.18	0.16	0.000174	***	0.03	1.77	0.05	0.04	0.04
976.97	**24.23**	0.9	1.88	0.86	0.85	0.0000203	***	14.56	0.28	2.26	0.24	0.23
999.58	**3.21**	0.77	1.39	0.94	1.3	0.000128	***	2.08	0.22	1.47	0.27	0.6
1015.11	**14.57**	1.48	2.11	1.26	1.09	0.0000124	***	8.3	0.65	1.92	0.73	0.4
1021.93	**2.45**	0.9	1.24	1.15	2.15	0.0000697	***	1.27	0.18	1.05	0.54	0.86
1167.86	3.27	1	1.45	1.35	**4.72**	<0.000001	***	1.76	0.56	1.03	1.1	2.23
1206.35	2.77	1.02	1.54	2.11	**5.47**	<0.000001	***	1.37	0.3	1.19	1.61	2
1215.62	0.91	1.27	**2.35**	1.18	1.51	0.000317	***	0.29	0.44	1.48	0.28	0.65
1253.27	0.89	1.26	**2.25**	1.27	1.65	0.000916	***	0.24	0.38	1.32	0.45	0.85
1429.52	**8.12**	1.36	1.69	1.44	1.56	0.000614	***	4.95	0.32	0.87	0.37	0.59
1575.42	**12.08**	1.52	2	2.28	1.27	0.0000124	***	7.37	0.26	1.5	1.73	0.23
1590.95	1.95	**2.89**	1.44	1.41	1.11	0.0000378	***	0.74	1.34	0.46	0.51	0.27
1613.65	**9.22**	1.3	1.84	1.69	1.51	0.0000124	***	3.82	0.2	0.67	0.66	0.57
2232.06	0.85	1.15	**1.95**	0.99	1.06	0.0000404	***	0.33	0.34	0.83	0.33	0.26
2586.1	0.77	4.09	**1.24**	0.98	**1.25**	0.000121	***	0.14	2.88	0.42	0.33	0.36
2899.02	0.62	**1.94**	0.97	0.72	1.1	0.000125	***	0.19	0.94	0.43	0.23	0.55
3017.62	0.66	**2.74**	0.85	0.84	0.88	0.000614	***	0.19	1.76	0.23	0.18	0.26
3047.83	0.6	**2.84**	1.02	0.96	1.02	0.000131	***	0.15	2.16	0.3	0.26	0.4
3132.42	0.51	**1.81**	0.96	0.85	0.77	0.0000509	***	0.11	1.07	0.31	0.26	0.2
3158.22	0.6	1.06	**3.45**	0.89	0.99	0.00011	***	0.12	0.47	4.7	0.27	0.25
3179.74	0.57	1.16	**2.07**	0.82	0.9	0.0000523	***	0.1	0.53	1.43	0.24	0.19
3195.13	0.51	0.97	**1.73**	0.89	0.78	0.000223	***	0.12	0.54	1.15	0.47	0.3
3261.26	0.57	1.06	**1.57**	0.81	0.78	0.0000203	***	0.1	0.64	1.01	0.23	0.14
3271.41	0.62	0.84	**1.73**	0.83	0.77	0.000327	***	0.2	0.29	1.48	0.26	0.2
3298.27	0.49	0.62	1.03	1.15	0.74	0.0000448	***	0.09	0.2	0.31	0.89	0.2
3352.67	0.68	1.19	**1.64**	1.24	0.82	0.0000523	***	0.41	0.46	0.67	0.72	0.23
3374.34	0.6	1.51	1.31	0.87	**2.45**	0.0000668	***	0.2	0.54	0.5	0.32	2.17
3384.76	1.64	**7.78**	1.14	1.33	1.49	0.000121	***	1.64	6.24	0.42	0.93	0.7
3403.29	0.78	**2.74**	1.12	1.36	1.3	0.0000697	***	0.29	1.56	0.42	0.77	0.43
3431.87	0.79	1.46	**2.81**	**2.94**	1.96	0.000334	***	0.34	0.32	2.63	2.49	1.44
3436.72	0.71	1.02	2.15	1.89	**3.1**	0.0000378	***	0.24	0.31	1.72	2.07	1.43
3438.51	0.66	1.01	2.39	1.61	**2.18**	0.000206	***	0.22	0.41	1.95	1.49	0.93
3639.29	0.51	**1.59**	0.72	0.69	0.72	0.0000697	***	0.08	1.09	0.19	0.19	0.19
4285.27	0.48	**1.58**	0.63	0.69	0.58	0.000786	***	0.09	0.9	0.15	0.21	0.18
4302.13	0.44	**1.64**	0.64	0.57	0.56	0.0000448	***	0.1	0.93	0.17	0.15	0.11
4438.42	0.59	**1.61**	0.58	0.52	0.52	0.000525	***	0.21	1.1	0.11	0.15	0.14
4525.99	0.46	**6.25**	0.61	0.59	0.6	0.00011	***	0.11	5.81	0.15	0.16	0.19
4570.82	0.57	**7.62**	0.72	0.57	0.6	0.000101	***	0.24	7.22	0.18	0.14	0.16
5849.97	**2.27**	0.44	0.41	1.14	0.49	0.0000448	***	2.02	0.1	0.1	1.37	0.32
6594.83	0.24	0.34	0.29	**0.78**	0.34	0.000267	***	0.05	0.08	0.09	0.69	0.11
6642.94	0.42	0.37	0.32	0.61	**1.44**	0.000359	***	0.26	0.11	0.11	0.32	1.41
6704.78	0.33	0.48	**0.84**	0.42	0.35	0.000469	***	0.23	0.21	0.64	0.21	0.1
6747.12	0.35	0.84	0.45	0.45	**1.33**	0.000317	***	0.16	0.43	0.12	0.17	1.34
6766.67	0.41	**6.57**	0.4	0.51	0.59	0.0000448	***	0.15	6.43	0.1	0.27	0.31
6783.1	0.38	**1.59**	0.4	0.59	0.56	0.0000883	***	0.14	1.09	0.1	0.3	0.22
6802.88	0.31	**1.81**	0.37	0.68	0.64	<0.000001	***	0.06	1.3	0.09	0.52	0.33
6870.39	0.3	0.48	1.28	1.29	**2.02**	0.0000124	***	0.16	0.14	1.39	2.09	1.21
6875.22	0.34	0.52	**1.69**	0.96	1.3	0.000585	***	0.08	0.19	1.84	1.15	0.65
779.16	1.25	**3.31**	1.93	2.96	0.9	0.00511	**	0.8	1.65	2.22	3.76	0.62
1038.77	1.33	1.94	**2.62**	1.25	2	0.0264	**	0.58	0.82	2	0.52	1
1069.38	0.81	0.93	**1.59**	0.99	0.98	0.0141	**	0.15	0.17	0.8	0.34	0.46
1925.67	0.87	1.09	1.23	1.08	**1.71**	0.0144	**	0.23	0.27	0.37	0.38	0.94
2341.58	0.61	1.02	1.02	**1.62**	0.93	0.00114	**	0.16	0.34	0.3	1.18	0.27
2678.79	0.55	0.73	0.88	**1.3**	0.8	0.00766	**	0.16	0.21	0.2	0.94	0.24
2903.64	0.68	1.12	**1.71**	0.88	1.26	0.00127	**	0.38	0.84	1.19	0.28	0.57
3285.62	0.63	0.89	**1.39**	0.73	0.76	0.04	**	0.26	0.87	1.23	0.23	0.26
3322.94	0.73	0.94	1.09	1.12	**2.18**	0.0106	**	0.46	0.49	0.51	0.58	1.67
3462.62	0.8	0.99	2.6	**1.55**	1.32	0.0133	**	0.31	0.22	2.59	1.38	0.58
3477.42	0.8	1.05	**2.09**	1.58	1.09	0.0176	**	0.5	0.39	1.82	0.94	0.47
3506.38	0.67	0.74	**1.95**	0.93	0.89	0.00127	**	0.24	0.18	1.75	0.29	0.2
3972.55	0.59	**1.91**	0.67	0.67	0.69	0.00492	**	0.23	0.99	0.23	0.19	0.18
4546.62	0.7	**1.57**	0.62	0.54	0.51	0.00408	**	0.59	1.09	0.2	0.15	0.15
4589.77	0.6	**1.87**	0.58	0.63	0.61	0.0147	**	0.29	1.2	0.14	0.15	0.16
4679.58	0.44	0.61	0.49	0.46	**0.78**	0.0199	**	0.21	0.21	0.18	0.17	0.43
4772.88	0.37	0.72	0.5	**0.88**	0.51	0.0026	**	0.12	0.26	0.13	0.74	0.15
5049.82	0.37	0.45	0.51	0.43	**0.93**	0.00331	**	0.05	0.09	0.14	0.15	0.62
5218.41	0.77	**2.48**	0.47	0.49	0.52	0.00126	**	0.37	1.79	0.12	0.16	0.14
5856.77	0.77	0.36	0.41	**1.34**	0.72	0.0224	**	0.34	0.11	0.12	1.69	0.87
6712.05	0.27	0.54	0.54	**0.62**	0.35	0.0144	**	0.13	0.39	0.33	0.55	0.16
6850.59	0.41	0.68	**1.25**	0.73	0.6	0.0229	**	0.16	0.34	0.93	0.71	0.21
6866.58	0.39	0.48	0.84	**1.79**	1.19	0.00186	**	0.15	0.07	0.67	2.62	1.08
6925.11	0.31	0.4	0.43	**0.93**	0.64	0.0304	**	0.17	0.1	0.19	1.15	0.52
757.12	0.42	0.52	0.48	0.81	0.48	0.0528		0.12	0.16	0.15	0.72	0.13
1527.78	0.4	0.77	0.78	1.94	0.15	0.646		0.63	0.91	1.32	2.72	0.27
1536.71	3.64	1.53	0.96	0.95	0.92	0.49		3.78	0.73	0.28	0.38	0.31
1659.96	4.56	0.59	0.46	0.45	0.43	0.627		6.11	0.24	0.1	0.14	0.11
1973.49	1.09	0.56	0.44	0.41	0.46	0.627		1.1	0.23	0.1	0.13	0.11
2011.74	0.37	0.44	0.47	1.14	0.38	0.451		0.09	0.12	0.16	1.44	0.09
2917.85	5.7	1.39	1.27	1.13	1.2	0.113		6.13	0.49	0.29	0.31	0.28
4849.59	1.59	1.86	2.3	2.38	1.52	0.649		0.73	0.45	2.26	1.7	0.39
5209.58	5.54	1.25	1.43	1.34	1.27	0.427		7.19	0.29	0.42	0.47	0.34
5501	11.69	1.38	1.38	1.27	1.25	0.45		16.56	0.31	0.42	0.26	0.37
5834.55	12.03	1.45	1.69	1.57	1.38	0.123		17.4	0.26	0.64	0.73	0.41

Appendix 1: Specifications for the genetic algorithm

ClinProt Model

Name:	**CBR_SNN6_5prot_pollen2311**
Date/Time:	2023-11-23T11:14:04.857+01:00
GUID:	5e064739-f266-4765-a525-6fdc516640b1
ClinProTools Version:	2.2 build 83

Model Generation Classes

Class 1:	E:\Spettri_polline\Polline_famiglie\Asteraceae
Class 2:	E:\Spettri_polline\Polline_famiglie\Boraginaceae
Class 3:	E:\Spettri_polline\Polline_famiglie\Fagaceae
Class 4:	E:\Spettri_polline\Polline_famiglie\Leguminosae
Class 5:	E:\Spettri_polline\Polline_famiglie\Rosaceae

Cross Validation

Percent Leave Out:	20 %
Number of Iterations:	10
**Overall:**	**80.81 %**
Class 1:	92 %
Class 2:	89.47 %
Class 3:	79.41 %
Class 4:	50 %
Class 5:	93.18 %

Recognition Capability

**Overall:**	**90.97 %**
Class 1:	100 %
Class 2:	84.62 %
Class 3:	83.33 %
Class 4:	91.67 %
Class 5:	95.24 %

ClinProt Model

Name:	**CBR_SNN6_5prot_pollen2311**
Date/Time:	2023-11-23T11:14:04.857+01:00
GUID:	5e064739-f266-4765-a525-6fdc516640b1
ClinProTools Version:	2.2 build 83

Model Generation Classes

Class 1:	E:\Spettri_polline\Polline_famiglie\Asteraceae
Class 2:	E:\Spettri_polline\Polline_famiglie\Boraginaceae
Class 3:	E:\Spettri_polline\Polline_famiglie\Fagaceae
Class 4:	E:\Spettri_polline\Polline_famiglie\Leguminosae
Class 5:	E:\Spettri_polline\Polline_famiglie\Rosaceae

Cross Validation

Percent Leave Out:	20 %
Number of Iterations:	10

**Overall:**	**80.81 %**
Class 1:	92 %
Class 2:	89.47 %
Class 3:	79.41 %
Class 4:	50 %
Class 5:	93.18 %

Recognition Capability

**Overall:**	**90.97 %**
Class 1:	100 %
Class 2:	84.62 %
Class 3:	83.33 %
Class 4:	91.67 %
Class 5:	95.24 %

Integration Regions used for Classification

Index	Mass	Start Mass	End Mass	Weight
77	6594.83	6589.86	6599.5	0.1526479576643106
17	1206.35	1204.75	1208.92	0.1043862706259356
52	3431.87	3428.27	3433.94	0.05320805450329364
51	3403.29	3398.22	3407.87	0.05199722315234185
35	2903.64	2902.38	2906.73	0.0514750991625243
16	1167.86	1166.62	1170.42	0.04490008317084811
3	787.62	784.14	793.34	0.04453786575141531
30	2232.06	2230.42	2236.71	0.04419081017168144
4	804.85	803.14	806.79	0.0402899976172942
84	6802.88	6792.13	6813.36	0.03800324911655435
26	1659.96	1656.46	1665.07	0.03569835290001412
7	830.6	829.26	836.51	0.03207840696153862
53	3436.72	3434.42	3438.03	0.03191951675644984
76	5856.77	5856.46	5865.07	0.02918160520297897
6	825.72	823.28	829.26	0.02901108974992941
15	1069.38	1067.05	1072.82	0.02774247787966494
43	3261.26	3259.91	3266.64	0.02699175716876981
10	976.97	972.38	982.82	0.02362503034498351
61	4302.13	4296.84	4305.35	0.02277085599124553
79	6704.78	6700.51	6708.48	0.02188741431449919
82	6766.67	6755.31	6778.75	0.01494716407985933
18	1215.62	1213.13	1219.18	0.01156642393589477
59	3972.55	3968.4	3978.48	0.0111169291258585
64	4546.62	4541.43	4549.57	0.009834351008775163

Resolution

Resolution:	800
Base Line:	top hat
Minimal Baseline Width:	10 %

Loading Spectra Collection

Data Reduction Filter:	false
Minimal Mass:	600
Maximal Mass:	18000
Null Spectra Exclusion:	true
Noise Spectra Exclusion:	false
Adduct/Polymer Spectra Exclusion:	false
Support Spectra Grouping:	true
Similarity Selection:	false

Recalibration

Recalibrate:	false

Average Peak List Calculation

Relative Threshold Base Peak:	0.0001
Signal to Noise Threshold:	5
Limit Peak Number:	true
Max Peak Number:	100
Peak Sort Mode:	signal to noise

Peak Calculation

Use Areas:	false
Peak List Edited Manually:	true

Peak Selection

Use All Peaks:	true
Sort Mode:	p value tta

Model Generation

Algorithm:	**SNN**
UpperLimit of Cycles:	2000
Automatic Number of Prototypes Detection:	false
Number of Prototypes:	5
